# Supplementary figures and images for: Sparse RNNs can support high-capacity classification
Source: PLoS Comput Biol. 2022 Dec 14;18(12):e1010759. doi: 10.1371/journal.pcbi.1010759 (PMC9797087; doi:10.1371/journal.pcbi.1010759)

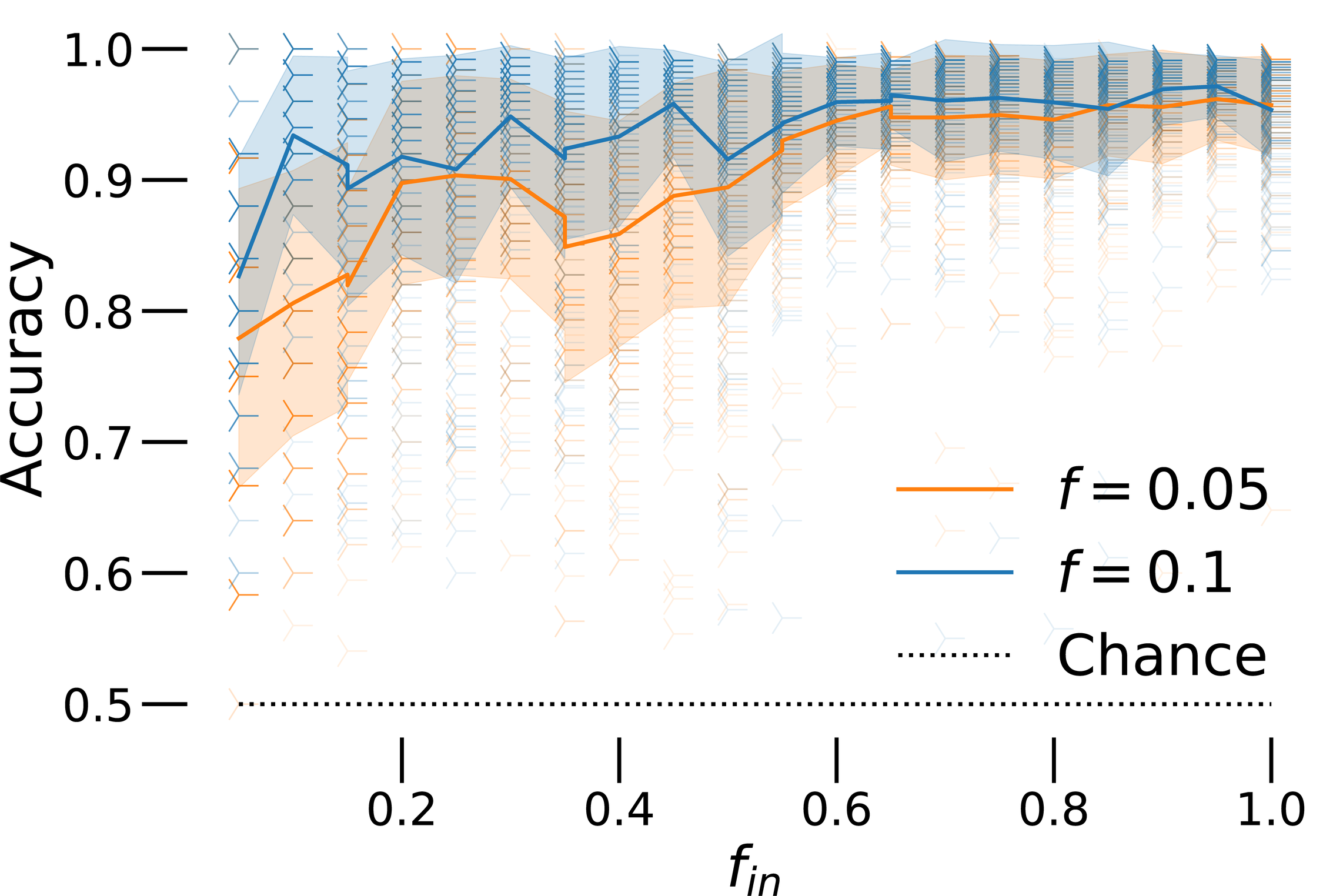

Supplement: S1 Fig — In addition to recurrent and output sparsity, we explored the effects of input connection sparsity. In this scenario, the number of patterns stored with good performance scales with the input sparsity as well, i.e. P = αfinfN2 instead of the result P = αfN2 we report throughout the rest of the paper. We report sparse RNN performance as a function of input sparsity (fin) for RNNs with two different levels of recurrent and readout sparsity (f). We have used P = αfinfN2. N = 100, α = 0.5, # epochs = 500, n = 160. (TIF) [file pcbi.1010759.s001.tif]

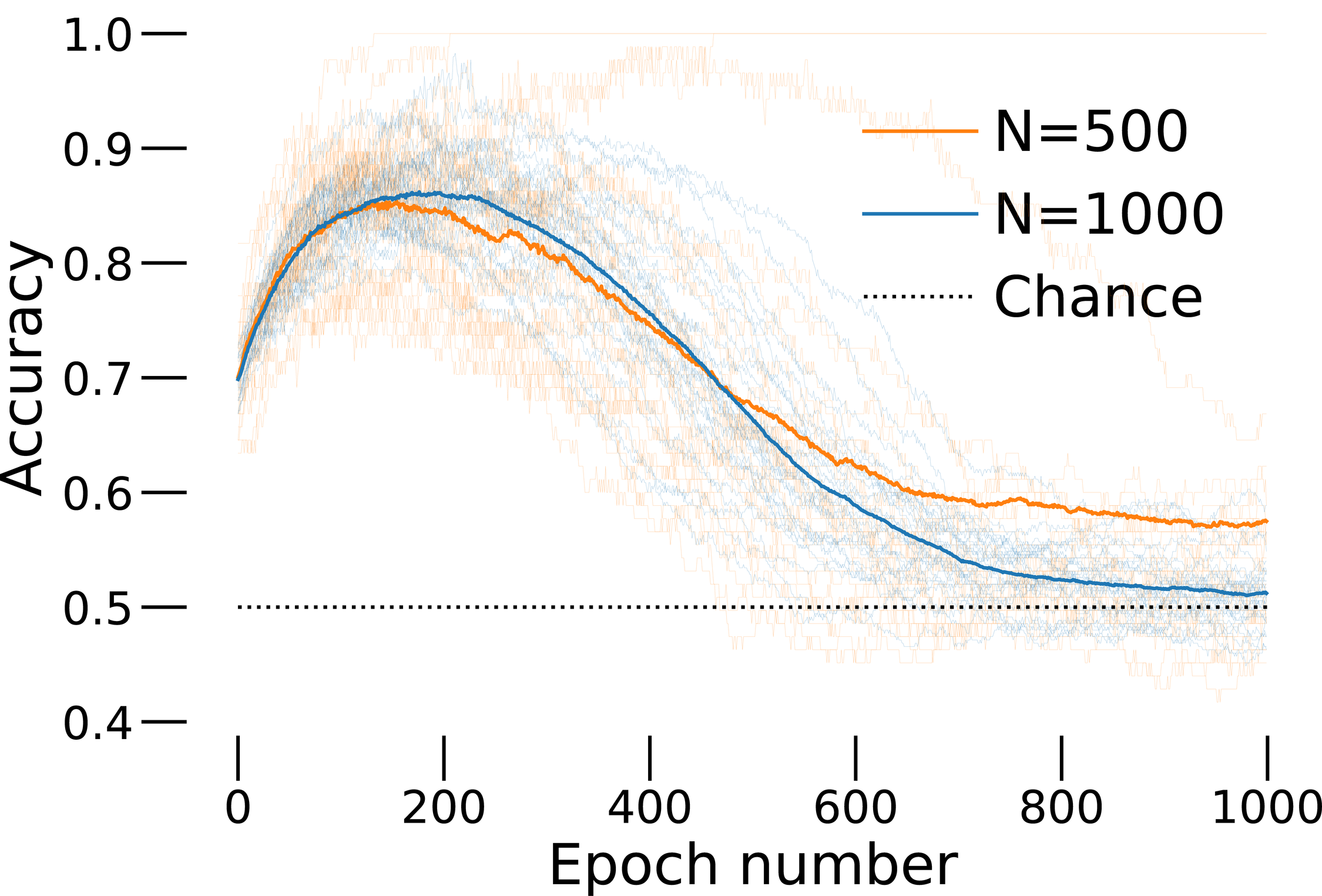

Supplement: S2 Fig — The OS+ Hebbian training method fails to maintain previously stored patterns when pushed beyond maximum capacity. We report sparse RNN performance as a function of the training epoch when trained with the OS+ method. Thick lines are averages and thin lines are individual simulations. f = 0.1, α = 0.007, n = 30. (TIF) [file pcbi.1010759.s002.tif]
